# SG-APSIC1040: Implementing a quality-improvement approach to surgical-site infection prevention in the Philippines

**DOI:** 10.1017/ash.2023.91

**Published:** 2023-03-16

**Authors:** Anthony Abustan, Arnel Francisco, Maria Teresa Andrade, Julie Winn, Paul Gwyn Pagaran, Ted Miles, Joan Littlefield, Unarose Hogan

**Affiliations:** Americares, Manila, Philippines; Camarines Norte Provincial Hospital, Daet, Philippines; Camarines Norte Provincial Hospital, Daet, Philippines; Americares, Stamford, Connecticut, United States; Americares, Manila, Philippines; Americares, Stamford, Connecticut, United States; Americares, Stamford, Connecticut, United States; Americares, Stamford, Connecticut, United States

## Abstract

**Objectives:** We aimed to reduce surgical site infections in Camarines Norte Provincial Hospital, Philippines, (1) by establishing SSI surveillance in the surgical departments, (2) by implementing quality improvement processes, and (3) by developing and implementing an SSI prevention care bundle. **Methods:** In partnership with Americares, SSI surveillance based on CDC criteria was instituted for all surgeries, excluding orthopedic surgeries. Staff were trained in applying quality-improvement methodology, infection prevention and control, and SSI prevention. A care bundle based on the WHO evidence-based interventions for SSI prevention was designed. Interventions included preoperative bathing, surgical hand preparation, intraoperative surgical-site preparation using 2% chlorhexidine isopropanol solution, and postoperative wound management. The model for improvement methodology was used to implement these changes for 12 months from May 2020 to May 2021. **Results:** In total, 718 surgeries were followed for SSI surveillance, with an average of 58 surgical patients per month in 2020, which increased to 90 patients per month in 2021. In 2020, the SSI incidence rate was 1.76%, and this rate increased 38.64% to 2.44% in 2021. A statistically significant increase in knowledge of 5.29 points (95% CI, 4.91–5.67) among 150 participants undergoing SSI training between pretest (+6.46) and posttest (+ 11.76) was achieved. SSI care-bundle checklists were used for 80% of eligible surgical patients by 2021. Compliance with the SSI care-bundle checklist increased from 0 to 87.69% (n = 718) by October 2021, subsequently decreasing by 2.75% by December 2021. **Conclusions:** A quality-improvement process embedded in routine surgical care can be a building block for reducing SSIs. However, we did not achieve an overall decrease in SSIs, likely due to increased reporting of SSIs through improved SSI surveillance. However, important gains were achieved in improved healthcare worker knowledge and practice through the implementation of an SSI care bundle. Fluctuations in checklist compliance reflected COVID-19 surges.

